# Ligand assisted growth of perovskite single crystals with low defect density

**DOI:** 10.1038/s41467-021-21934-6

**Published:** 2021-03-16

**Authors:** Ye Liu, Xiaopeng Zheng, Yanjun Fang, Ying Zhou, Zhenyi Ni, Xun Xiao, Shangshang Chen, Jinsong Huang

**Affiliations:** 1 Department of Applied Physical Sciences, University of North Carolina, Chapel Hill, NC 27599 USA; 2 Department of Mechanical and Materials Engineering, University of Nebraska-Lincoln, Lincoln, NE 68588 USA

**Keywords:** Materials for devices, Optical materials and structures

## Abstract

A low defect density in metal halide perovskite single crystals is critical to achieve high performance optoelectronic devices. Here we show the reduction of defect density in perovskite single crystals grown by a ligand-assisted solution process with 3‐(decyldimethylammonio)‐propane‐sulfonate inner salt (DPSI) as an additive. DPSI ligands anchoring with lead ions on perovskite crystal surfaces not only suppress nucleation in solution, but also regulate the addition of proper ions to the growing surface, which greatly enhances the crystal quality. The grown CH_3_NH_3_PbI_3_ crystals show better crystallinity and a 23-fold smaller trap density of 7 × 10^10^ cm^−3^ than the optimized control crystals. The enhanced material properties result in significantly suppressed ion migration and superior X-ray detection sensitivity of CH_3_NH_3_PbI_3_ detectors of (2.6 ± 0.4) × 10^6^ µC Gy^−1^air cm^−2^ for 60 kVp X-ray and the lowest detectable dose rate reaches (5.0 ± 0.7) nGy s^−1^, which enables reduced radiation dose to patients in medical X-ray diagnostics.

## Abstract (web summary)

The performance of a metal halide perovskite single crystal is governed by the defect density. Here, the authors report a high quality single crystal perovskite grown by a ligand-assisted solution process with DPSI achieving 23-fold smaller trap density than that without DPSI.

## Introduction

Metal halide perovskite (MHP) has become a rising star semiconductor candidate for ionizing radiation detection, due to its superior and unique optoelectronic properties, such as large carrier mobility, long carrier recombination lifetime^1,2^, high defect tolerance^3^, and high stopping power to ionization radiation^4–6^. Perovskite single crystals are an important set of materials not only as the platform for the study of their intrinsic physical properties^7^, but also to achieve record-performance devices such as solar cells^8^, photodetector^9^, and ionization radiation detectors^6,10^. Single crystals have shown better physical properties than polycrystalline materials such as larger mobility, longer recombination lifetime, less ion diffusion^11^, less un-intentional doping^12^, and better stability^13^, which are contributed to the absence of extended defects such as grain boundaries as well as the lower point defect density^14^. To grow MHP single crystals, solution-based methods are still simplest, and low-cost in making perovskite single crystals of different compositions with large quantity. Many efforts have been devoted to grow perovskite single crystals of different compositions and shapes to optimize their device performance. The growth rates of different facets were tailored by choosing the non-stoichiometry of raw material or different solvents, which resulted in the crystals in different shapes^15–17^. Thin crystals with a large aspect ratio were grown on solution/air interface^18–20^ or between two substrates with space confinements^21,22^. All solution-based single crystal growth methods create supersaturated solution so that solute can precipitate out. Among them, crystal growth through inverse temperature crystallization (ITC) method is particularly attractive, which utilizes the reduced solubility of perovskites in different solutions at a range of higher temperature^23–25^. To obtain large-sized bulk single crystals, the single crystal was carefully transferred as seed to another fresh precursor for multiple cycles^26^ or using oil to continuously extract solvents^27^. However, there are much less efforts devoted to enhancing the quality of perovskite single crystals in terms of reducing point defect density, despite it is well known in the community that the crystals quality is important to achieve the best properties and device performance.

In this work, we introduced coordinating organic molecules in perovskite precursor solution as additives to regulate the growth of perovskite single crystals which impact both nucleation and crystal growth processes. The additives with strong interaction to lead ions in perovskites can tune the growth rate of different facets, and more importantly reduce the point defect formation. The obtained single crystals with improved quality showed a smaller defect density by 23 folds, which dramatically suppressed ion migration and improved optoelectronic properties. The application of these high quality CH_3_NH_3_PbI_3_ (MAPbI_3_) single crystals for medical X-ray detection achieved the record high sensitivity of (2.6 ± 0.4) × 10^6^ µC Gy^−1^air cm^−2^ and lowest detectable dose rate of (5.0 ± 0.7) nGy s^−1^ for 60 kVp X-ray.

## Results and discussion

### Additive modification of crystal growth

**Fig. 1:**
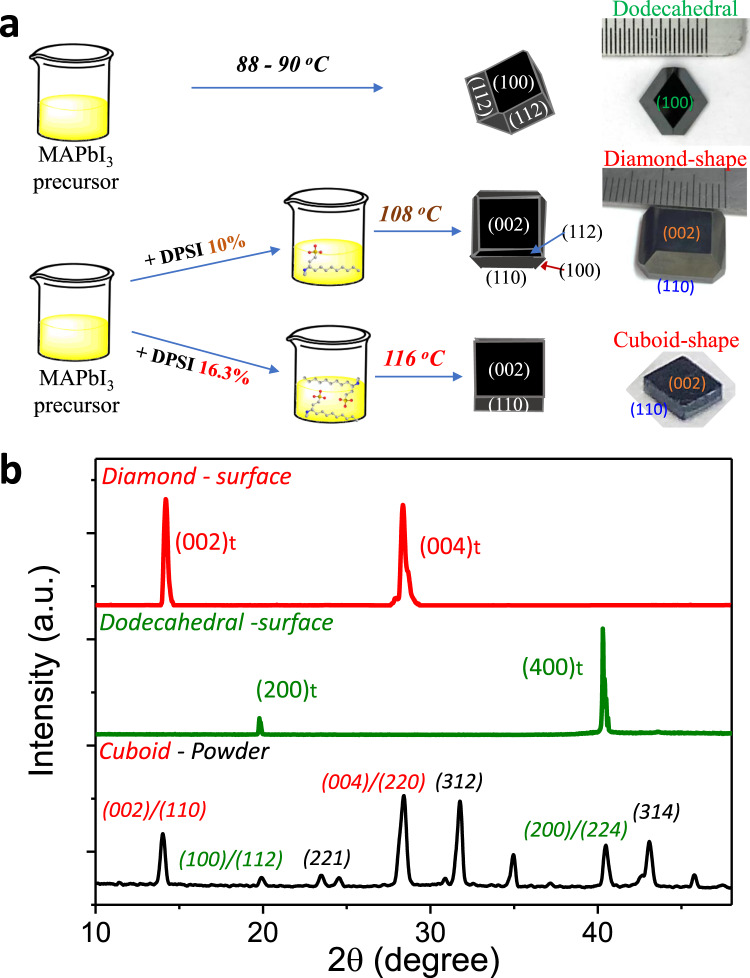
Crystal growth behavior with ligand regulation. **a** Scheme of crystals growing process and conditions, and photographs of the grown crystals. **b** XRD patterns of the facets of the crystals and powder of the cuboid-shape crystal.

Like any crystal growth process, there is a trade-off between growth speed and crystal quality. A higher growth temperature is beneficial to reduce the crystal defect density, because the precursor ions have a larger diffusion rate so that they can find the right lattice position to stick to. There are always chances that point defects can be generated due to the absence of an ion or a right type of ion showing up at the lattice position, which may cause the formation of point defects like vacancies or antisites, even at high temperature. Reducing crystal growth speed to decrease defect formation is not always an option in applications. Our method of changing crystal growth kinetics and reducing defect density is to introduce some organic molecules which have strong interaction with ions on the surface of perovskite crystals to regulate the addition of precursor ions onto the crystals. Such molecules should have interaction with ions of perovskites to some extent, but the interaction should not be too strong to form primary chemical bonding permanently, which would hinder or terminate crystal growth. This design rule indicates that most organic molecules which are broadly used for defect passivation in perovskite solar cells can act on this role. Here we chose one molecule previously studied for surface passivation on MHP thin film solar cells, 3‐(Decyldimethylammonio)‐propane‐sulfonate inner salt (DPSI), because it not only passivates defects in MHP polycrystalline grains, but also modulates the grain size in the polycrystalline films^28^. The MAPbI_3_ single crystals were grown using the ITC method where crystals precipitate from MAPbI_3_ precursor solutions after they were heated to a growth temperature which is dependent on the precursor concentration^25,29^. The MAPbI_3_ single crystal grown from control precursor solution without DPSI was dodecahedral shape, and exposed facets are (100)t and (112)t, where t donates tetragonal phase, as shown in Fig. 1a. After adding 10% molar ratio of DPSI to Pb in the precursor solution, the grown crystals had more cuboid shape with facets of (002)t, (110)t, and (112)t. A single crystal with dimensions of 12.9 mm × 12.6 mm × 3.7 mm was obtained from this precursor with 10% DPSI at 108 °C after growing for 50 h (Fig. 1a). Further increasing the DPSI ratio to 16.3% resulted in disappearance of the higher index plane of (112) and thus completely cuboid shape crystals (Fig. 1a). Here the facets of these crystals were determined by X-ray diffraction (XRD) measurement, as shown in Fig. 1b and Supplementary Fig. 1. The powders diffraction pattern of the MAPbI_3_ cuboid-shape crystal grown with 16.3 mol% DPSI shows it is still tetragonal phase (Fig. 1b). It is noted that the required nucleation temperature was 18–20 °C higher, while the growth temperature only needed 8 °C higher after pre-seeding to reach the same growth rate (Supplementary Fig. 2). To test whether this method is general in controlling crystal growth, DPSI was then added into the single crystal growth of a different composition of MAPbBr_3_. As shown in Supplementary Fig. 3a, b, DPSI also reduced the growth rate of MAPbBr_3_, and a higher growth temperature was required for crystal growth with same rate.

### Mechanism of crystal growth using DPSI additive

**Fig. 2:**
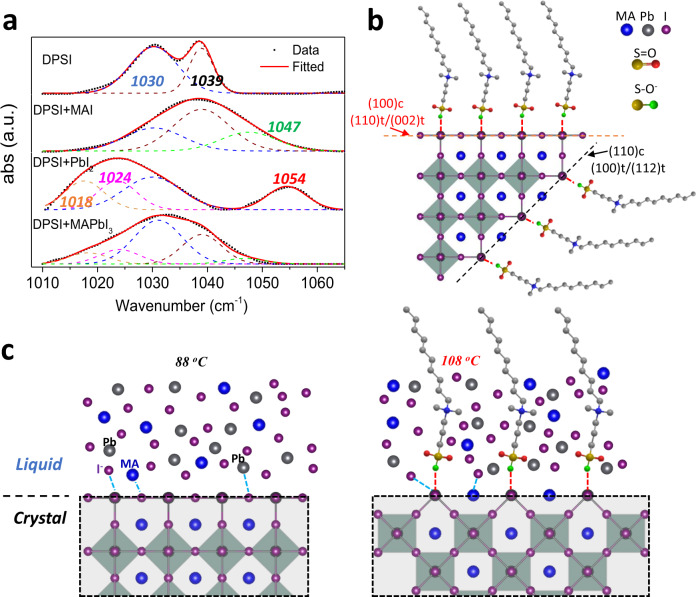
Mechanism of crystal growth with DPSI. **a** FTIR spectra of the powders of MAI, PbI_2_, and MAPbI_3_ with 10 mol% DPSI. **b** Scheme of DPSI absorption difference on different crystal facets which have different Pb^2+^ density. **c** Scheme of crystal growth mechanism at the surface of crystal where the DPSI molecules modulate the diffusion of metal ions to reach the perovskite surface.

To understand the interaction of DPSI with perovskites, the Fourier transform infrared spectroscopy (FTIR) measurements were conducted after mixing MAPbI_3_ and DPSI. In Fig. 2a, for pristine DPSI powder (after vacuum drying), the peaks of the sulfonic acid group at 1030 cm^−1^, 1039 cm^−1^ were observed^30,31^. The two peaks are assigned as S–O^-^ (1030 cm^−1^) and S = O (1039 cm^−1^) in -SO_3_^−^ group of DPSI. To further understand the interaction between DPSI and MAPbI_3_, FTIR characterization was also applied onto the mixtures of the PbI_2_:DPSI, MAI:DPSI and MAPbI_3_:DPSI, respectively, which were prepared by dissolving them in solvents and drying the solution. The peak shifts of -SO_3_^−^ group in FTIR spectra of the three samples were compared with that of pristine DPSI powder. A fitted peak of 1047 cm^−1^ in MAI:DPSI sample suggests that MA^+^ forms hydrogen bond with S = O in DPSI. For PbI_2_:DPSI sample, the S–O^-^ peak of DPSI red-shifted, and it can be fitted into two new peaks of 1018 cm^−1^ and 1024 cm^−1^, which indicates there are more than one interaction scenarios of Pb^2+^ with S–O^-^. The shifting of S = O from 1039 cm^−1^ to 1054 cm^−1^ suggests a coordination interaction of the lone pair electrons of oxygen in S = O with Pb. The S–O^-^ peak in MAPbI_3_:DPSI is dominantly by the red-shifted 1030 cm^−1^ peak, and the main fitted peaks are still 1018 cm^−1^ and 1024 cm^−1^, which are caused by the interaction of S-O^-^ with Pb^2+^. The hydrogen bonds of S = O in DPSI with MA are almost negligible and the ionic bonding of -SO_3_^−^ with Pb^2+^ dominates the interaction of DPSI and MAPbI_3_, as illustrated in Fig. 2b.

The strong interaction of the S–O^-^ group of DPSI with Pb^2+^ indicates that the surfaces of perovskite crystals in solution can be wrapped by DPSI. The steric hindrance effect from the long -(CH_2_)_n_- chain of the DPSI, working as the polymer brushes with a thickness of 1-2 nm^32^, would hinder the diffusion of ions to the perovskite surfaces for attachment, as illustrated in Fig. 2c. The hindrance effect of DPSI would change the growth rate of different facets^33,34^. We calculated the Pb atom density at different facets. As shown in Table 1, Pb atom density on (110)t and (002)t is largest, and thus these facet would grow slower and survive. The suppression of crystal growth at (110)t and (002)t facets were directly observed when DPSI was added with different weight percentages into the precursor solution after seeding with regular dodecahedral-shaped crystals (Supplementary Fig. 2a). The (112) facet with a lower density of Pb ions would grow much faster, eventually resulting in its disappearance (Fig. 1a and Supplementary Fig. 2a)^35^ To maintain the same crystal growth rate, the crystal growth temperature needed to be increased from 88 °C to 96 °C, as shown in Supplementary Fig. 2b. We speculate that an elevated temperature would also enhance the dynamic detaching of DPSI to the perovskite surfaces, which allows more solute ions to diffuse and attach to crystal surfaces, as illustrated in Fig. 2c. The increased crystal growth temperature also enhanced the diffusion rate of ions in the precursor solution, which allows the right ions to be delivered timely to the surface lattice position and thus reduces the defect density in the grown single crystals^36,37^. This could not be achieved by simply enhancing the crystal growth temperature in regular precursors without DPSI, because an increased temperature generally causes the formation of a larger quantity of nucleus everywhere in the solution. A too large density of nucleus in solution would quickly consume the solute in the solution, and they would attach to other growing crystals, which prevents the crystals growing into large size, as demonstrated by the growth of MAPbBr_3_ in Supplementary Fig. 3a. Therefore, another main function of DPSI is to suppress the nucleation, as evidenced by the less crystals in the solution with DPSI in Supplementary Fig. 3). Since the temperature needs to be 20 °C (for MAPbI_3_ with DPSI) higher than normal to reach the same nucleation rate, while the same crystal growth rate can be achieved by increasing temperature by only 8 °C. DPSI additive enables a new crystal growth temperature window to achieve the same growth rate but with suppressed nucleation. Finally, the interaction of Pb^2+^ or undercoordinated-Pb with DPSI also introduces an energy barrier for the diffusing ions for further crystal growth. Only if the binding energy of incoming species with Pb is stronger than Pb-DPSI under high growth temperature, such as I^−^Pb bonding, DPSI would be replaced and the crystal can keep growing. This mechanism can effectively increase the diffusion barrier and the defect formation energy to reduce the possibility of bonding or stacking of wrong ions.

**Table 1:** Calculated atomic area density in different facets of typical MAPbI_3_ crystals in tetragonal phase.

Facets	Pb atomic density (atoms Å^−2^)	I atomic density (atoms Å^−2^)
{002}t	0.02548	0.05096
{110}t	0.02522	0.05044
{100}t	0.01824	0.01824
{112}t	0.01793	0.01793

### High quality single crystals

**Fig. 3:**
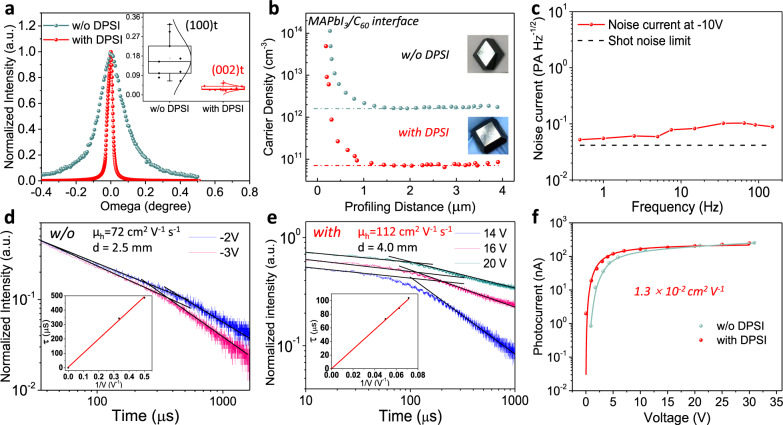
Material and electronic characterization of MAPbI_3_ crystals. **a** X-ray rocking curves for MAPbI_3_ single crystals grown w/o and with DPSI, and the statistical FWHM results are shown as the insert. **b** Trap density profiled by DLCP for the crystals at different profiling distance from top surface. **c** Noise current of the crystal grown with DPSI. **d**, **e** Time of flight transient currents of the crystals grown w/o and with DPSI. **f** Photoconductivity comparison of the crystal w/o and with DPSI under weak ambient light intensity of 1 × 10^−4^ W cm^−2^.

The changed crystal growth kinetics of MAPbI_3_ single crystal grown with DPSI additive should also affect the crystal crystallinity. To investigate whether DPSI introduces a crystallinity difference, we conducted X-ray rocking curve measurements on these crystals. Here nine samples from each type of crystals were studied for a statistical comparison. The photographs of some crystals with DPSI in different size are shown in Supplementary Fig. 4. As shown in Fig. 3a, the average full width at half maximum (FWHM) of the rocking curve for (002) facet was 0.0289° for the crystals grown with DPSI, while that value was 0.1650° for the crystals grown without additives, and its distribution is also much broader (insert of Fig. 3a). This small FWHM of 0.0199° for the champion crystal grown with DPSI almost reached the resolution of our equipment, and is among the best quality MAPbI_3_ single crystals reported so far. Such a sharp rocking curve for the MAPbI_3_ single crystals grown with DPSI confirmed that the crystallinity has been dramatically improved. Normally the crystal with improved crystallinity will has a lower defect density. We then measured the charge trap density caused by defects using drive-level capacitance profile (DLCP) technique, which was recently shown to characterize the spatial distribution of defect densities in perovskite single crystals^14^. Figure 3b shows the depth dependent trap densities for deep traps with a depth of > 0.43 eV from the top surface (C_60_/MAPbI_3_ interface) to the interior for the as-grown crystals without any surface treatment. It is noted these crystals still have a large density of defects at very top surfaces, because there was some precursor residual on the crystal surfaces when the crystals were removed out of precursor solution. The trap density at the interior of MAPbI_3_ crystals grown with DPSI reached 7 × 10^10^ cm^−3^, which is the smallest directly measured trap density among all perovskite single crystals reported in literature. The deep trap density in the MAPbI_3_ crystals grown with DPSI is about 23 times smaller than the control MAPbI_3_ crystal, directly proves that DPSI additive can reduce the defect density in the single crystals. This shows that high-quality single crystals with reproducible good crystallinity can be obtained without sacrificing the growth rate. The presence of charge traps would generally cause a frequency dependent noise (pink or 1/f noise with a high noise a low frequency) due to the charge trapping and detrapping process^38–40^. To evaluate the impact of the reduced defect trap density on the device noise, we directly measured it by a Fast Fourier Transform (FFT) signal analyzer and a low noise current preamplifier within the frequency range from 0.5 Hz to 150 Hz at bias of −10 V. Figure 3c shows that the noise current of the crystal grown with DPSI was almost independent of frequency, which again supports the very low defect density in the crystal.

The hole carrier mobility of the MAPbI_3_ crystals was also compared by time of flight (TOF) method. As shown in Fig. 3d, e, the hole carrier mobility in MAPbI_3_ crystal grown with DPSI was largely improved from 72 cm^2^ V^−1^ s^−1^ to 112 cm^2^ V^−1^ s^−1^. We also compared the MAPbBr_3_ single crystals to see if DPSI can also improve crystal quality. As shown in Supplementary Fig. 5, the hole mobility of the MAPbBr_3_ crystal with DPSI was slightly increased from 132 cm^2^ V^−1^ s^−1^ to 144 cm^2 ^V^−1^ s^−1^, while the electron carrier mobility increased dramatically from 65 cm^2^ V^−1^ s^−1^ to 224 cm^2^ V^−1^ s^−1^. The carrier mobility-lifetime product (µτ) was derived by fitting the photocurrent of the MAPbI_3_ devices with modified Hecht equation. As shown in Fig. 3f, the µτ of MAPbI_3_ crystal was enhanced from 8.4 × 10^−3^ cm^2^ V^−1^ to 1.3 × 10^−2^ cm^2^ V^−1^ after growth with DPSI under ambient light with an intensity of 1 × 10^−4^ W cm^−2^, one of the highest reported values for MHPs.

**Fig. 4:**
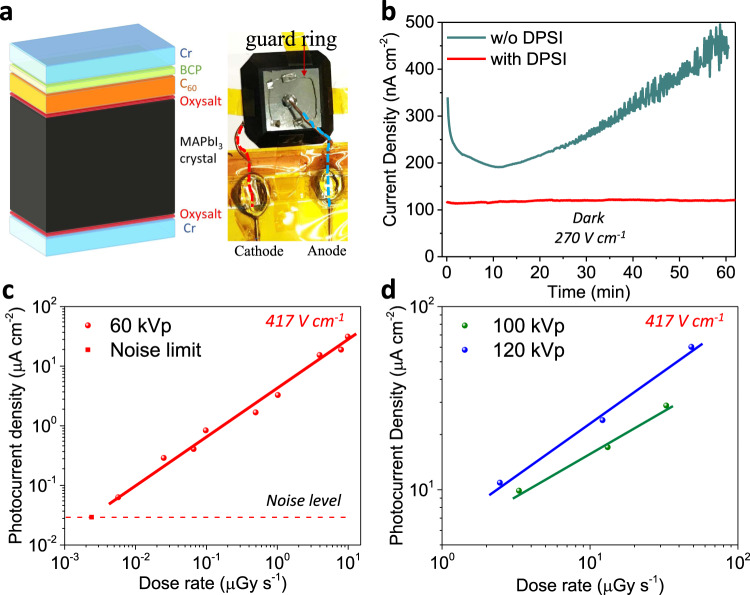
X-ray detection performance. **a** Device structure scheme and a photo of the as-fabricated device. **b** Dark current density of the crystals under a same electric field of 270 V cm^-1^ at ambient air without encapsulation. The crystals were both polished and passivated by oxysalt. The thickness of the crystal with DPSI here is 3.7 mm, and the applied bias was −100 V. **c** Device output current under 60 kVp X-ray of different dose rate with noise limit. **d** Device output current under 100 kVp and 120 kVp X-rays. This crystal thickness is 2.4 mm, and the applied bias was −100 V.

It is noted that not all defects cause electronic charge traps, and some structural defects may cause doping or facilitate ion migration^12,14^. The iodine-based MHP materials are promising candidates in X-ray detection, due to the high atomic number, large attenuation coefficient, and high photogenerated carrier density^6,41^. However, the most widely studied MHPs for X-ray radiation detectors were mainly bromine-based compositions for their low noise level^42–45^. Iodine-based perovskites were reported to have low resistivity and obvious ion migration under bias^6,46^. Ion migration-related current drift or polarization effect has been a major concern for the application of MHPs for radiation detection. It is noted that the fastest ion migration occurs through point defects or extended defects. We thus speculate the perovskite single crystals with a much low defect density should be much more stable under electric field. To evaluate the stability and quality improvement of the single crystal by adding DPSI, we applied a bias for an elongated time to track the dark current drift^43^, since dark current drift has been shown to be a good indicator of ion migration or polarization effect in many type of single crystal materials^47,48^. The as-grown MAPbI_3_ single crystals with DPSI have been fabricated into detector devices with guard ring^42^ and the structure of Cr (20 nm) /MAPbI_3_/C_60_ (30 nm)/ bathocuproine (BCP, 8 nm)/Cr (20 nm). As shown in Supplementary Fig. 6 and Supplementary Table 1, the dark current drift of these high-quality crystals without any surface treatment or passivation under different electric field reached 3.72 ± 2.23 × 10^−5^ nA cm^−1^ v^−1^ s^−1^, which represents the lowest dark-current drift for iodine-based MHP devices. Since surface defects still exist at the perovskite crystal surface, as suggested by DLCP results, we further polished all MAPbI_3_ single crystals to remove the surface residuals and treated the exposed surface with (C_8_H_17_NH_3_)_2_SO_4_ solution for ~2 s at room temperature. And then the crystals were annealed at 50 °C for 5 min, which induces the formation of a robust, wide bandgap oxysalt to passivate the crystal surface dangling bonds^49^. These treatments can slow down the surface degradation under bias which would otherwise cause electrochemical reaction of perovskite with metal electrodes. Device structure of the crystals after surface treatments is shown in Fig. 4a. As shown in Fig. 4b, the crystal grown with DPSI is not only more intrinsic due to the smaller dark current of ~48 nA (dark current density of 120 nA cm^−2^) under a field of 270 V cm^−1^, but also shows more than 10 times less dark current drift than pristine crystal during 1 h continuous test (Fig. 4a). The dark current density drift of the MAPbI_3_ crystal device with DPSI under such a high bias was 1.2 × 10^−5^ nA cm^−1^ v^−1^ s^−1^ (Table 2), which is comparable to the best reported inorganic perovskites Cs_2_AgBiBr_6_ device^43^. It is known that I^-^ ions are much more mobile than Br^−^ ions^50^, therefore the small dark current drift in MAPbI_3_ crystal devices indicates a significant smaller iodine-related defect density. The resistivity of the MAPbI_3_ crystal detector with DPSI under different electric field was measured to be 1.95 ± 0.35 × 10^9^ Ω cm (Table 2), which is among the highest of iodide-based perovskite and also suggests their low defect density, since defect can cause self-doping^14,51,52^.

**Table 2:** Dark current drift and resistivity result of the MAPbI_3_ crystal with DPSI after surface treatment in Fig. 4b.

Reverse bias (V)	Electric field (V cm^−1^)	Dark current (nA cm^−2^)	Resistivity (Ω cm)	Current drift (nA cm^−1^ v^−1 ^s^−1^)
5	13.5	8.7	1.55 × 10^9^	1.6 × 10^−6^
10	27	15.6	1.73 × 10^9^	6.58 × 10^−6^
20	54	34.5	1.56 × 10^9^	1.3 × 10^−5^
100	270	120	2.25 × 10^9^	1.2 × 10^−5^

### Application of crystals for X-ray detection

Finally, we measured the X-ray detection performance using these high-quality MAPbI_3_ crystals. Firstly, the X-ray detector based on the crystal of Fig. 4b with DPSI has shown excellent sensitivity of 2.1 × 10^5^ µC Gy^−1^air cm^−2^ with lowest detectable dose rate of 2.34 nGy s^−1^ under soft X-ray energy with 8 keV (Supplementary Fig. 7). However, in medical imaging, hard X-rays with energy above 60 kVp are mostly used for a high penetration capability, so the hard X-rays with energy of 60 kVp, 100 kVp and 120 kVp were used in this study with their simulated energy spectrum shown in Supplementary Fig. 8. Under bias of −100 V on a crystal with thickness of 2.4 mm (electric field of 417 V cm^−1^), the dark current was measured about 81 nA (current density of 2.71 × 10^3^ nA cm^−2^) with 4.7 × 10^−5^ nA cm^−1^ v^−1 ^s^−1^ drifted under continuous tracking. This detector delivered high sensitivities of 2.9 × 10^6^ µC Gy^−1^air cm^−2^ for 60 kVp X-ray (Fig. 4c), 6.01 × 10^5^ µC Gy^−1^air cm^−2^ for 100 kVp X-ray, and 1.04 × 10^6^ µC Gy^−1^air cm^−2^ for 120 kVp (Fig. 4d). The lowest detectable dose rate for 60 kVp X-ray reached 5.7 nGy s^−1^ (Fig. 4c). This is among the best X-ray detection performances in all MHP crystal detectors regardless of compositions, as summarized in Supplementary Table 2. The low noise of these high quality MAPbI_3_ single crystals allows them to compete with bromine-based and low-dimension MHPs, such as MAPbBr_x_Cl_3-x_^42^, Cs_2_AgBiBr_6_^43^, (NH_4_)_3_Bi_2_I_9_ (2D crystal)^44^, given that their lower bandgap and higher atomic number of I than Br should allow the generation of a larger density of charge carriers under X-ray.

In this work, we have significantly improved the quality of MAPbI_3_ single crystals by ligand regulation during crystal growth, with 23 times lower bulk defect density, without sacrificing crystal growth rate. The ion migration under high bias has been successfully suppressed for the first time for iodide perovskites, which is comparable to inorganic perovskites. The crystal detectors achieve ultra-high sensitivity of (2.6 ± 0.4) × 10^6^ µC Gy^−1^air cm^−2^ and the lowest detectable dose rate of 5.0 ± 0.7 nGy s^−1^ to 60 kVp X-ray.

## Methods

### Materials and crystal growth

The solvents of γ-butyrolactone (GBL, > 99%), N,N-Dimethylformamide (DMF, > 98%), lead bromide (PbBr_2_, > 98%), BCP (96%), and DPSI were all purchased from Sigma-Aldrich. MAI and MABr powder were purchased from GreatCell Solar. Lead iodide (PbI_2_, 99.9985%) was purchased from Alfa Aesar. All the MHP single crystals were grown by typical ITC method respectively. C_60_ ( > 99.5%) was purchased from Nano-C and Cr was purchased from Kurt J. Lesker. For MAPbI_3_ single crystals in this study, MAI and PbI_2_ powder were mixed in molar ratio of 1:1 with concentration of 1.5 M in GBL to prepare the same initial precursor solution. For MAPbBr_3_ single crystals, MABr and PbBr_2_ powder were mixed in molar ratio of 1:1 with concentration of 1.2 M in DMF to prepare the initial precursor. The oxysalt passivation solution was synthesized with the same method^49^ and diluted with the concentration of 1 mM in mixed solvents (toluene/isopropanol = 5:1).

### Characterizations

XRD patterns were carried out on a Rigaku MiniFlex 6 G system with an X-ray tube at 2.0 kW. This was configured with Cu Kα radiation (wavelength of 1.5418 Å). The medium resolution X-ray rocking curve measurements were conducted by a Rigaku Smartlab X-ray diffractometer equipped with a 3 kW Cu tube at V = 40 kV and I = 44 mA. The parallel beam geometry was applied to achieve medium resolution parallel X-ray source, which is further confined with a 10 mm slit. The step size for omega scan is 0.0001 degree. The FTIR spectra of the powder samples were scanned from 4000 cm^−1^ to 400 cm^−1^ and recorded on a PerkinElmer FTIR spectrometer in reflection mode. All the mixture samples of PbI_2_:DPSI, MAI:DPSI, and MAPbI_3_:DPSI for FTIR were prepared by drying out their blended solution to study their interactions.

### Capacitance measurements

The DLCP measurements were performed by using an Agilent E4980A precision LCR meter. The scanning range of the ac frequency (*f*) was 0.02–2000 kHz. The demarcation energy $E_\omega = kT\,{\mathrm{ln}}\left( {\frac{{\omega _0}}{\omega }} \right)$ (where *ω*_0_ is the attempt-to-escape angular frequency that equals to 2*πv*_0_*T*^2^ is derived from the temperature-dependent *C*–*f* measurements. The *ν*_0_ is obtained from the fitting of ${\mathrm{ln}}\left( {\frac{{T^2}}{\omega }} \right) = \frac{{E_T}}{{kT}} - \ln \,\left( {2\pi v_0} \right)$ obtained at different *T*. Temperature-dependent capacitance measurements were carried out in a Lake Shore Cryotronics probe stage with a Lake Shore Cryotronics temperature controller model 336. SMA connected cables and ZN50R DC/RF probes that were applicable for RF test up to 1 GHz were equipped with the probe station. Before each measurement, the system was self-calibrated under open-circuit and short-circuit conditions to compensate for any undesired signal from the instrument.

For the DLCP measurements, the dc bias (*V)* was scanning from 0 V to the *V*_OC_ (*e.g*., 1.1 V) for the single crystal detectors. While ac bias (*δV)* was ranging from 20 to 200 mV. The capacitance measured at each *δV* was recorded and fitted with a polynomial function *C* = *C*_0_ + *C*_1_*δV* + *C*_2_(*δV*)^2^ + …to obtain *C*_0_ and *C*_1_. With the determination of *C*_0_ and *C*_1_, the total carrier density (*N*) that includes both free carrier density and trap density at the profiling distance *X* from the junction barrier is calculated by $N = - \frac{{C_0^3}}{{2q\varepsilon A^2C_1}}$, where *q* is the elementary charge, *ε* is the dielectric constant of the semiconductor and *A* is the active area of the junction. The profiling distance from the junction barrier was calculated by *εA*/*C*_0_, which was changed by tuning the *V*. For each ac bias, an additional offset dc voltage was applied to keep the maximum forward bias constant. The trap density within a certain trap depth range was calculated by subtracting the total carrier density measured at a larger *E*_ω_ (lower ac frequency) with that measured at a smaller *E*_ω_ (higher ac frequency).

### Time of flight (TOF) measurement

ToF measurement was conducted by illuminating the devices with 4 ns width, 337 nm laser pulses (SRS N2 laser) from the semitransparent electrodes side. The pulse laser-generated photocurrent was recorded using an Agilent 1 GHz digital oscilloscope (Agilent DSO-X 3104 A).

### X-ray radiation detection measurement

The continuous X-ray source for 60 kVp, 100 kVp and 120 kVp energy detection measurements was manufactured by YXLON with a 21-mm Al sheet filter and focal spot of 1 mm. The different current of the X-ray source and different thickness of 1100 Aluminum alloy sheets were applied to tune the dose rate values during measurement. The dose rate calibration above 60 nGy s^−1^ was measured by Raysafe X2 Solo dosimeter and using the same calibration process as our previous work^41^. The dose rate calibration below 60 nGy s^−1^ was measured by SOEKS Quantum radiation detector dosimeter same as our previous method^42^.

### Noise current measurement

Noise current was carried out at different frequency by a Fast Fourier Transform (FFT) signal analyzer (Agilent 35670 A) which was connected to a low noise current preamplifier.

## Supplementary information

Supplementary Information

## Data Availability

The data that support the findings of this study are available from the corresponding author upon reasonable request.
