# Supplementary material for: Ligand assisted growth of perovskite single crystals with low defect density
Source: Nat Commun. 2021 Mar 16;12:1686. doi: 10.1038/s41467-021-21934-6 (PMC7966356; doi:10.1038/s41467-021-21934-6)
Supplement: Supplementary file 1 — Supplementary Information [file 41467_2021_21934_MOESM1_ESM.pdf]

Supplementary Information

for

Ligand Assisted Growth of Perovskite Single Crystals with Low Defect Density

Ye Liu[1,2], Xiaopeng Zheng[2], Yanjun Fang[2], Ying Zhou[1], Zhenyi Ni[1], Xun Xiao[1], Shangshang Chen[1], Jinsong Huang[1*]

[1]Department of Applied Physical Sciences, University of North Carolina, Chapel Hill, NC 27599, USA.

[2]Department of Mechanical and Materials Engineering University of Nebraska-Lincoln, Lincoln, Nebraska 68588, USA

Supplementary Table 1. Dark current drift and resistivity of the as-grown $MAPbI_3$ crystals with DPSI in Fig. S6 without any surface treatment.

Electric field ($V\ cm^{-1}$)	Dark current ($nA\ cm^{-2}$)	Resistivity (Ω cm)	Current drift ($nA\ cm^{-1}\ v^{-1}s^{-1}$)
50	27.2	1.84×10^{9}	5.95×10^{-5}
125	48.8	2.56×10^{9}	2.45×10^{-5}
250	80	3.13×10^{9}	1.48×10^{-5}

Supplementary Table 2. Summary of the sensitivity and lowest detectable dose rate of different perovskite X-ray direct and indirect radiation detectors.

	Perovskite composition	X-ray energy (keV or kVp)	Sensitivity ($\mu C\ Gy^{-1}air\ cm^{-2}$)	Lowest detectable dose rate ($\mu Gy\ air\ s^{-1}$)	Dark current drift ($nA\ cm^{-1}\ v^{-1}\ s^{-1}$)	References
Indirect conversion (Scintillators)	$MAPbCl_3$ single crystal	50		0.1147		4
	Ru_2CuBr_3	30		0.1215		5
	$Rb_3Bi_2I_9$	50	159.7 (1 V bias)	0.00832	1.82×10^{-7}	6
	$MAPbBr_3$ single crystal	8	80	0.5		1
	$MAPbBr_3$/Si integration	8	2.1×10^{4}	0.036	1.2×10^{-3}	3
	$MAPbBr_xCl_{3-x}$ single crystal	8	8.4×10^{4}	0.0076		2
	$MAPbBr_3$ single crystal (oil growth)		184.6			7
	$(NH_4)_3Bi_2I_9$-		8.2×10^{3}	0.055		8

Direct conversion (Radiation detectors)	2D single crystal					
	BiOBr passivated $Cs_2AgBiBr_6$ wafer film	50	250 (0.5V μm^{-1})	0.0953	7.4×10^{-5}	9
	$Cs_2AgBiBr_6$ single crystal	30	105	0.0597		10
	$MAPbI_3$ cuboid shape crystal		968.9			11
	(GMA)$MAPbI_3$ single crystal	8	2.3×10^{4} (5V bias)	0.0169		12
	$MAPbI_3$ sintered wafer	70	2.527×10^{3} (200V bias)		1.7×10^{-3}	13
	$MAPbI_3$ pressed sinter	40	1.22×10^{5} (10 V bias)	2.54		14
	$Cs_3Bi_2I_9$ Single crystal	40	1.652×10^{3} (60 V bias)	0.130		15
	$(F\text{-}PEA)_2PbI_4$ 2D single crystal	120	3.402×10^{3} (200V bias)	0.023	4.9×10^{-8}	16
	$MAPbI_3$ (printable film)	100	1.1×10^{4}			17
	$MAPbI_3(Cl)$ in membrane	60	8,696±228			18
		100	1.44×10^{4} (20V bias)			
	$MAPbI_3$ thin single crystal (co-planar)	50	7.0×10^{5} (5V bias)	0.0015		19
		8	2.1×10^{5} (10V bias)	0.00234	6.58×10^{-6}	

	$MAPbI_3$ single crystal with ligand-assisted		2.8×10^5 (20V bias)		1.3×10^{-5}	This work
		60	2.9×10^6 (100V bias)	0.0057	4.7×10^{-5}	
		100	6.51×10^5 (100V bias)			
		120	1.04×10^6 (100V bias)			

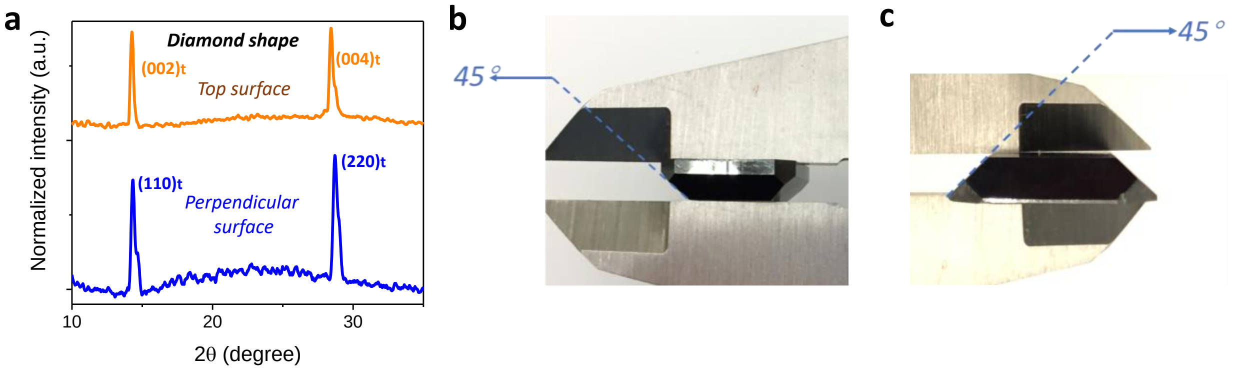

Supplementary Fig. 1. a) XRD pattern of different facets of the diamond-shaped crystal shown in Fig.1A. **b, c)** photos of the two crystals grown in 10% DPSI added, and the facet angles were measured.

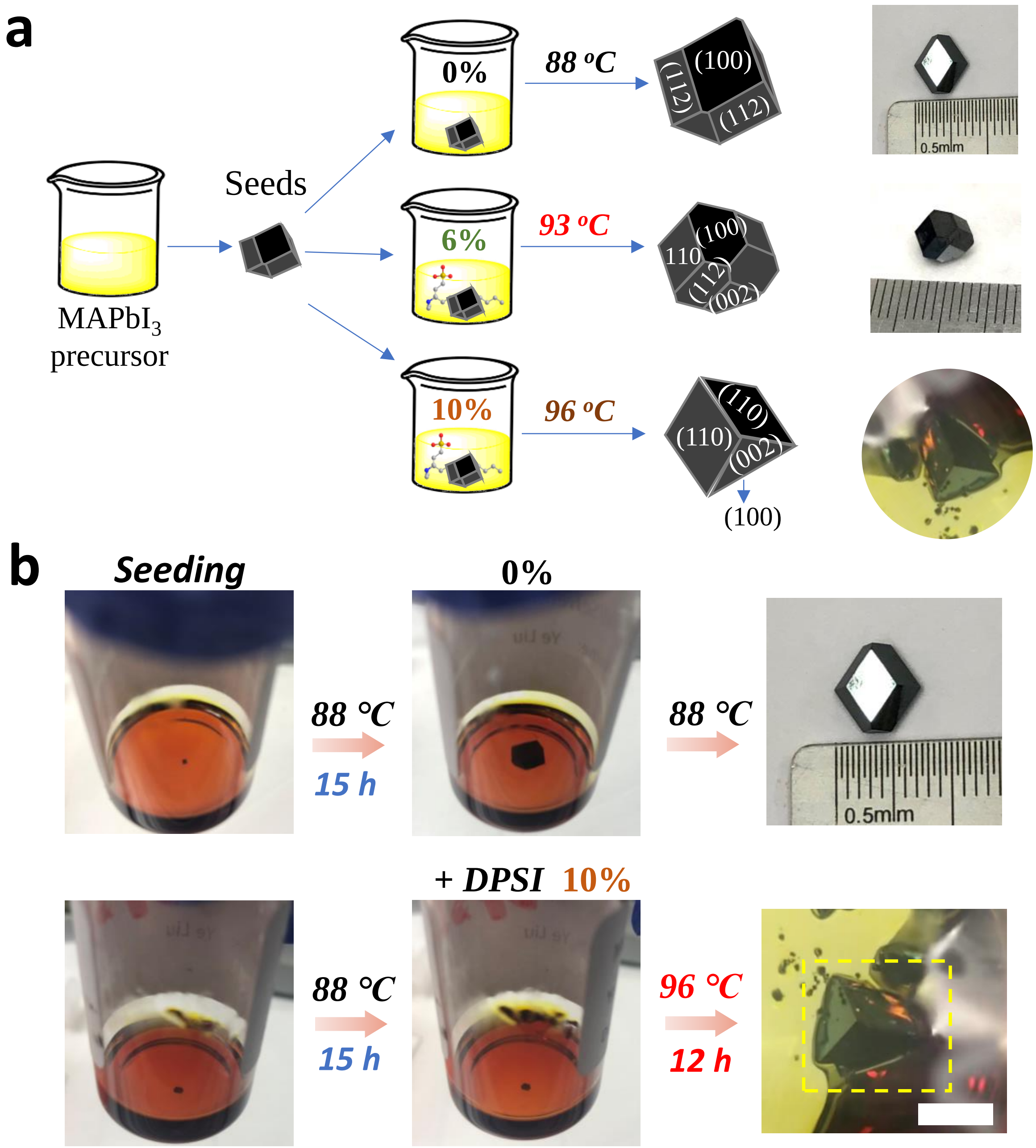

Supplementary Fig. 2. Crystal growth behavior with ligand regulation after seeding. a) Scheme and photos of crystal growth process after seeding dodecahedral crystals (pristine $MAPbI_3$ crystal) in precursor solutions w/o and with DPSI. **b)** The crystal growth rate comparison of the crystals w/o and with DPSI at different temperature after seeding. The scale bar is 5 mm.

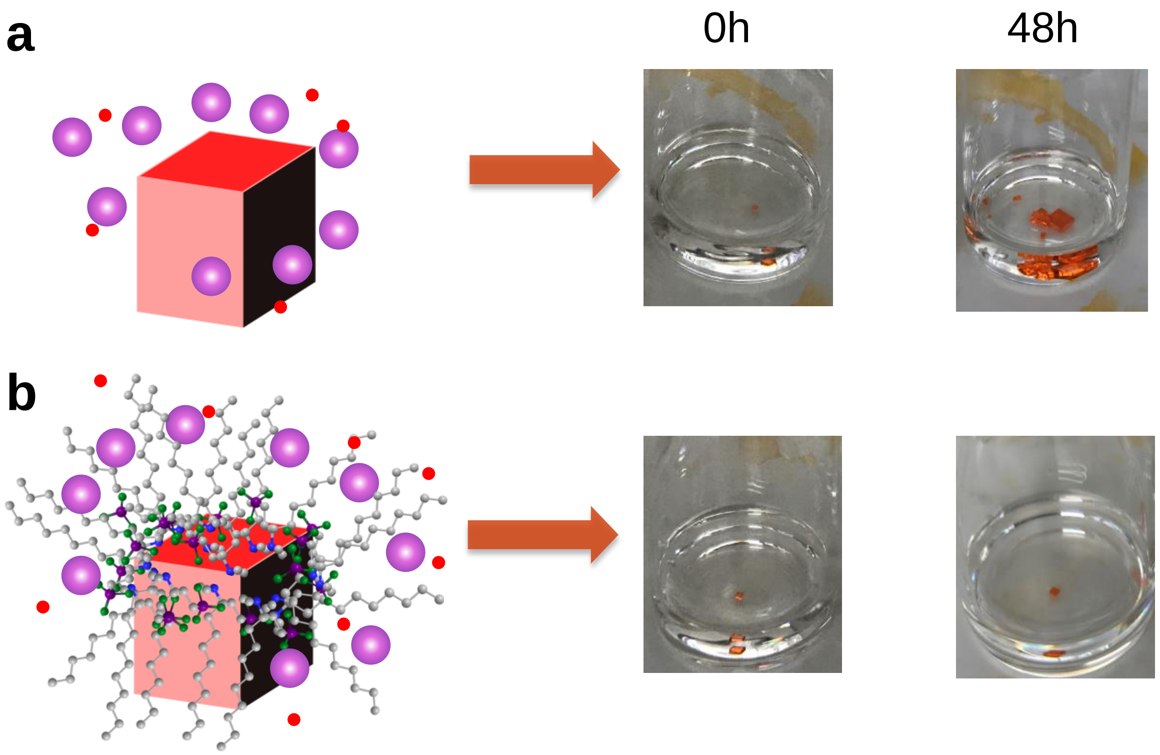

Supplementary Fig. 3. Crystal growth behavior of $MAPbBr_3$ single crystals with adding DPSI in precursor. a, b) Scheme and photograph of the crystals growth rate comparison w/o and with DPSI.

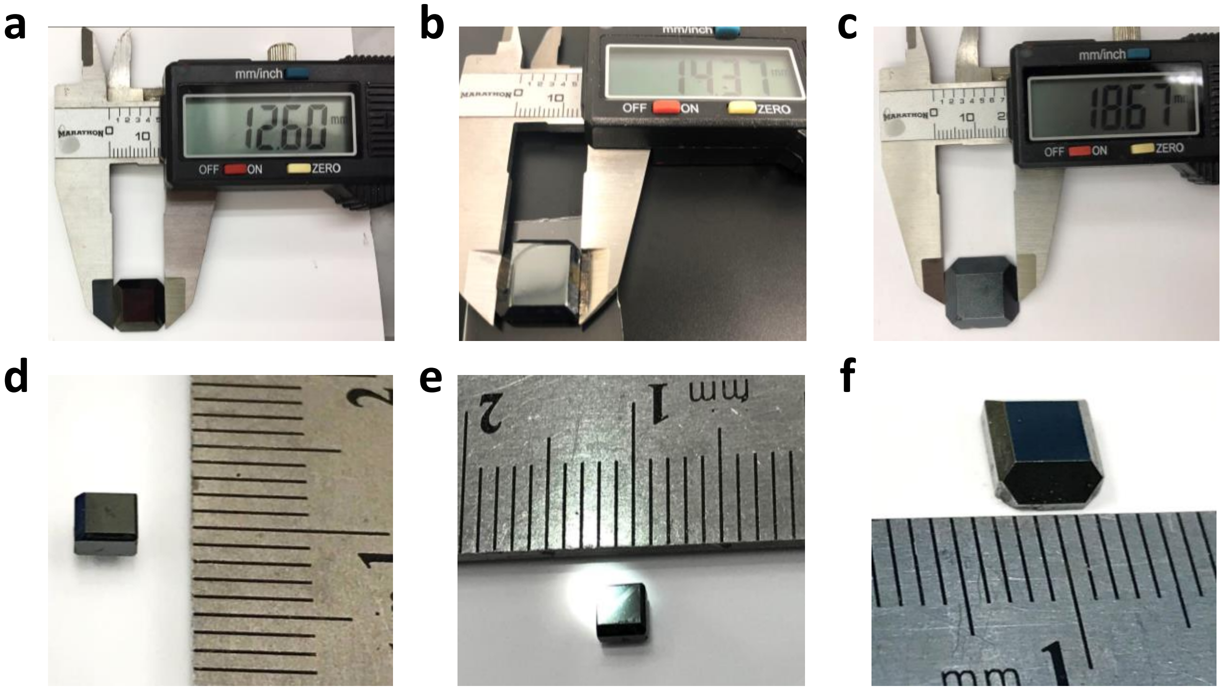

Supplementary Fig. 4. Photographs of $MAPbI_3$ single crystals grown with molar ratio of 10% DPSI in different dimensional sizes: a-c) some large crystals above 1 cm in length and **d-f)** some small crystals.

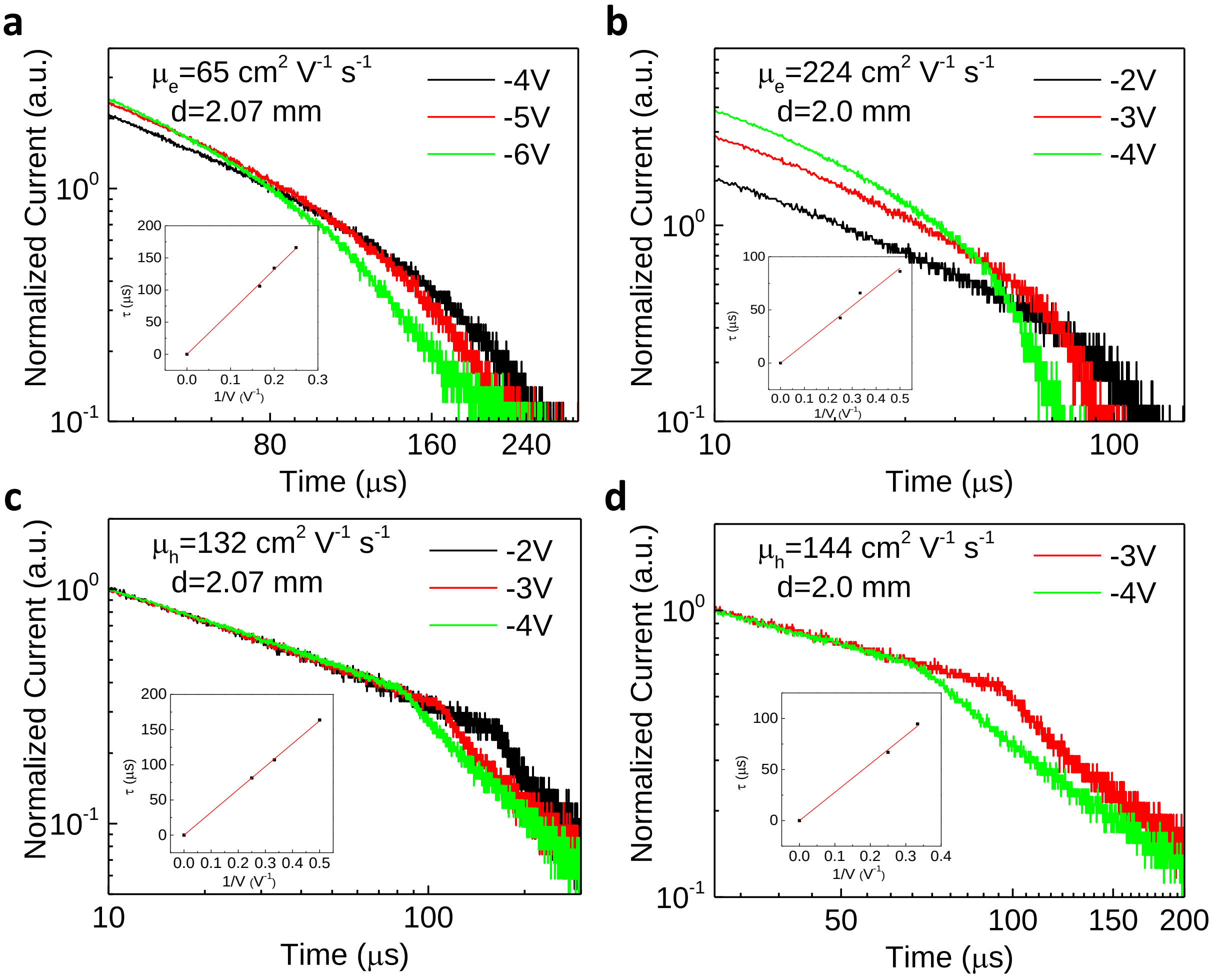

Supplementary Fig. 5. Crystal quality improvement of $MAPbBr_3$ single crystals with DPSI in precursor solution. Electron and hole carrier mobility of the crystals grown **a, b**) w/o and **c, d)** with DPSI, respectively.

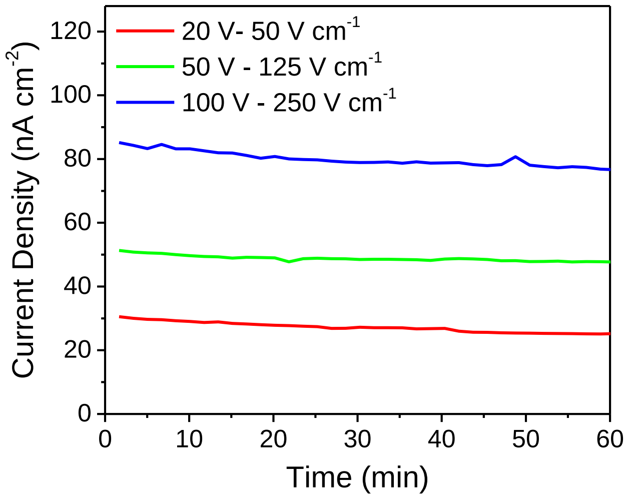

Supplementary Fig. 6. Dark current drift of a non-encapsulated device based on $MAPbI_3$ single crystal grown with DPSI under different applied electric field of 50 V cm^{-1}, 125 V cm^{-1} and 250 V cm^{-1}. The crystal thickness was 4 mm without any surface treatment before device fabrication, and the photograph of the crystal was shown in Supplementary Fig. 4c.

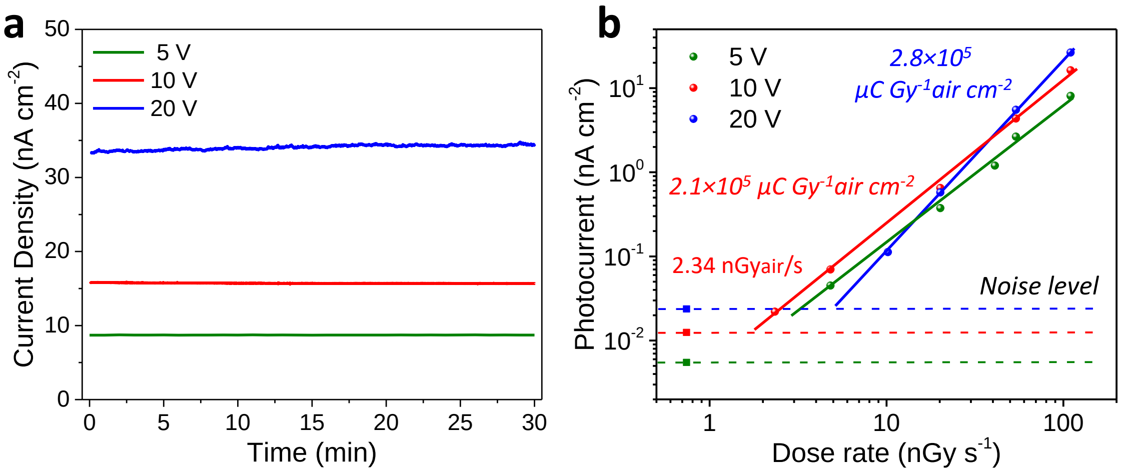

Supplementary Fig. 7. Performance of the X-ray detector under soft X-ray energy with energy of 8 keV. a) Dark current drift in air without encapsulation, and **b)** X-ray sensitivity measurement excited by 8 keV X-ray beam energy under bias of -5 V, -10 V and -20 V. The

crystal thickness was 3.7 mm, which is the same crystal in Fig. 4b and its photograph is shown in Supplementary Fig. 4a.

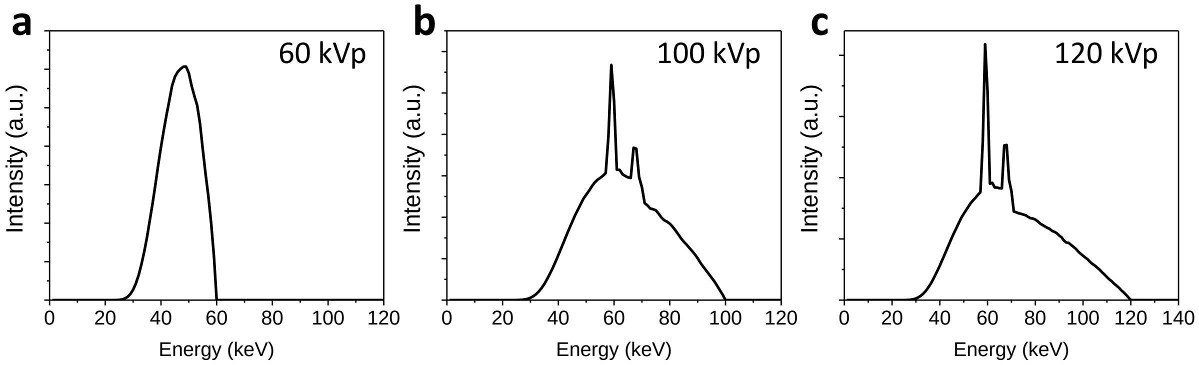

Supplementary Fig. 8. Simulated X-ray energy spectrum of the source by SPEKTR 3.0 with 21 mm Al filter at **a)** 60 kVp, **b)** 100 kVp and **c)** 120 kVp.

Supplementary References

1. Wei, H. *et al.* Sensitive X-ray detectors made of methylammonium lead tribromide perovskite single crystals. *Nature Photonics* **10**, 333-339 (2016).

2. Wei, H. *et al.* Dopant compensation in alloyed $CH_3NH_3PbBr_{3-x}Cl_x$ perovskite single crystals for gamma-ray spectroscopy. *Nature materials* **16**, 826-833 (2017).

3. Wei, W. *et al.* Monolithic integration of hybrid perovskite single crystals with heterogenous substrate for highly sensitive X-ray imaging. *Nature Photonics* **11**, 315-321 (2017).

4. Xu, Q. *et al.* High-sensitivity X-ray imaging of a lead halide perovskite single-crystal scintillator. *Optics Letters* **45**, 355-358 (2020).

5. Yang, B. *et al.* Lead-Free Halide Rb_2CuBr_3 as Sensitive X-Ray Scintillator. *Advanced Materials* **31**, 1904711 (2019).

6. Xia, M. *et al.* Unveiling the Structural Descriptor of $A_3B_2X_9$ Perovskite Derivatives toward X‐Ray Detectors with Low Detection Limit and High Stability. *Advanced Functional Materials* **30**, 1910648 (2020).

7. Yao, F. *et al.* Room-temperature liquid diffused separation induced crystallization for high-quality perovskite single crystals. *Nature communications* **11**, 1-9 (2020).

8. Zhuang, R. *et al.* Highly sensitive X-ray detector made of layered perovskite-like $(NH_4)_3$ Bi_2I_9 single crystal with anisotropic response. *Nature Photonics* **13**, 602-608 (2019).

9. Yang, B. *et al.* Heteroepitaxial passivation of $Cs_2AgBiBr_6$ wafers with suppressed ionic migration for X-ray imaging. *Nature communications* **10**, 1-10 (2019).

10. Pan, W. *et al.* $Cs_2AgBiBr_6$ single-crystal X-ray detectors with a low detection limit. *Nature photonics* **11**, 726-732 (2017).

11. Ye, F. *et al.* High‐quality cuboid $CH_3NH_3PbI_3$ single crystals for high performance X‐ray and photon detectors. *Advanced Functional Materials* **29**, 1806984 (2019).

12. Huang, Y. *et al.* A‐site Cation Engineering for Highly Efficient $MAPbI_3$ Single‐Crystal X‐ray Detector. *Angewandte Chemie International Edition* **58**, 17834-17842 (2019).

13. Shrestha, S. *et al.* High-performance direct conversion X-ray detectors based on sintered hybrid lead triiodide perovskite wafers. *Nature Photonics* **11**, 436-440 (2017).

14. Hu, M. *et al.* Large and dense organic–inorganic hybrid perovskite $CH_3NH_3PbI_3$ wafer fabricated by one-step reactive direct wafer production with high X-ray sensitivity. *ACS applied materials & interfaces* **12**, 16592-16600 (2020).

15. Zhang, Y. *et al.* Nucleation-controlled growth of superior lead-free perovskite $Cs_3Bi_2I_9$ single-crystals for high-performance X-ray detection. *Nature communications* **11**, 1-11 (2020).

16. Li, H. *et al.* Sensitive and Stable 2D Perovskite Single‐Crystal X‐ray Detectors Enabled by a Supramolecular Anchor. *Advanced Materials* **32**, 2003790 (2020).

17. Kim, Y. C. *et al.* Printable organometallic perovskite enables large-area, low-dose X-ray imaging. *Nature* **550**, 87-91 (2017).

18. Zhao, J. *et al.* Perovskite-filled membranes for flexible and large-area direct-conversion X-ray detector arrays. *Nature Photonics* **14**, 612-617 (2020).

19. Song, Y. *et al.* Atomistic Surface Passivation of $CH_3NH_3PbI_3$ Perovskite Single Crystals for Highly Sensitive Coplanar-Structure X-Ray Detectors. *Research* **2020** (2020) .
